# Adequacy and quality of abdominal echographies requested by primary care professionals

**DOI:** 10.1186/1471-230X-10-101

**Published:** 2010-09-06

**Authors:** Ma Antònia Auladell, Llorenç Caballeria, Guillem Pera, Lluís Rodríguez, José Dario Casas, Jesús Aznar, Dolores Miranda, Carmen Sánchez, Antonio Negrete, Josep Ma Castellví, Jesús Bernad, Santiago Canut, Josep Aubà, Miren Maite Aizpurua, Pere Torán

**Affiliations:** 1 Primary Healthcare Centre Premià de Mar, Catalan Health Institute, IDIAP Jordi Gol, La Plaça 93, 08330 Premià de Mar, Spain; 2 Primary Healthcare Research Support Unit Metropolitana Nord. IDIAP Jordi Gol, Camí del Mig 36, 08303 Mataró, Spain; 3 Radiology Department, Primary Healthcare Badalona, Catalan Health Institute, C/Pl. de la Medicina s/n, 08911 Badalona, Spain; 4 Radiology Department, Primary Healthcare El Maresme, Catalan Health Institute, Camí del Mig 36, 08303 Mataró, Spain; 5 Radiology Department, Primary Healthcare Santa Coloma de Gramanet, Catalan Health Institute, C/Major, 49-53, 08921 Santa Coloma de Gramanet, Spain; 6 Primary Healthcare Centre Gatassa, Catalan Health Institute, Camí del Mig 36 (4a planta), 08303 Mataró, Spain; 7 Digestive Service, Hospital de Mataró, Consorcio Sanitario del Maresme, C/Carretera Cirera s/n, 08304 Mataró, Spain; 8 Primary Healthcare Centre Vilassar de Mar, Catalan Health Institute, C/Santa Maria 59-79, 08340 Vilassar de Mar, Spain; 9 Primary Healthcare Centre Vilassar de Dalt, Catalan Health Institute, C/Plaça de la Vila 8, 08339 Vilassar de Dalt, Spain; 10 Primary Healthcare Barcelonés Nord i Maresme, Catalan Health Institute, Sardana s/n, 08915 Badalona, Spain

## Abstract

**Background:**

The value of abdominal echography in primary care is great because it is innocuous, inexpensive, easy to perform and provides a great deal of information making this the first examination to be requested in cases of probable abdominal disease. **However, too many abdominal echographies are probably requested overcrowding the Departments of Radiodiagnosis with not always justified petitions or with repetition of tests based on little clinical criteria**.

**Methods/Design:**

**The aim of the study is **to evaluate the adequacy and quality of abdominal echographies requested by primary care physicians in the Maresme County (North of Barcelona), develop guidelines for indicating echographies and reevaluate this adequacy after implementing these guidelines.

We will perform a two-phase study: the first descriptive, and retrospective evaluating the adequacy and quality of petitions for abdominal echographies, and in the second phase we will evaluate the impact of recommendations for indicating abdominal echographies for PC physicians on the adequacy and quality of echography petitions thereafter.

**This study will be carried out in 10 primary care centres **in the Maresme (Barcelona).

1067 abdominal echographies requested by primary care physicians from the above mentioned centres from January 2007 to April 2010 and referred to the Department of Radiology and the same number of applications after the intervention.

All the petitions for abdominal echographies requested will be analysed and the clinical histories will be obtained to determine demographic variables, the reason for the visit and for the echography petition and diagnostic orientation, clinical and echographic data, evaluation of the echographies according to the quality and variables characterising the professionals requesting the echographies including: age, sex, laboral situation, length of time in work post, formation, etc.

To achieve a consensus of the adequacy of abdominal echography, a work group including gastroenterologists, radiologists and general practitioners will be created following the nominal group. This will allow the design of guidelines for the indication of abdominal echography and posterior evaluation of their impact among physicians by diffusion and posterior reevaluation of the adequacy of the petitions.

## Background

Hepatic echography is widely used to study liver diseases and the biliary tract. The technique is inexpensive, is performed within a few minutes, has no secondary effects and does not require special preparation, except for fasting in studies of the gall bladder.

Echography is the most indicated technical tool in terms of cost-effectiveness for the initial study of all patients suspected of having hepatobiliary involvement due to the presence of hepatomegaly, ascites, jaundice, alterations in liver function tests, or pain in the upper right quadrant, etc. [1].

In addition, echograhy is the technique of choice for early detection of hepatocellular carcinoma in hepatic cirrhosis or in the follow up of patients who must undergo repeated sequential studies (response to treatment, liver transplantation) since periodic repetition of the echographic examination has no contraindications [2].

The sensitivity of echography in the diagnosis of diffuse lesions varies (approximately 70% in hepatic cirrhosis), correctly identifying well established steatosis and cirrhosis as well as the signs of advanced portal hypertension. In the differential diagnosis of ictericias this technique distinguishes dilatation of the biliary tract in 98% of the cases. It is, however, not as useful in the aetiological diagnosis of obstruction [3,4]. Its precision in the diagnosis of biliary lithiasis is of approximately 97%, also being very useful in the diagnosis of its complications. Echography is also indicated in the diagnosis of acute abdomen [5].

Advances in the use of echography have allowed it to become the optimum method for directing not only diagnostic punctures (fine needle puncture of tumours, cysts or abscesses and biopsy puncture for the diagnosis of diffuse liver diseases) [2] but also therapeutic punctures (sclerosing treatment of tumours, radiofrequency, abscess drainage and cholecystectomies) [6,7].

Thus, abdominal echography constitutes an excellent examination in most cases and should be recommended as an appropriate test whenever possible.

Our primary and hospitalary health care system presents a good level of health care which has led to increasing requests for complementary tests aimed at achieving satisfactory clinical diagnoses. On the other hand, health care pressure is very high with the consequent overcrowding of both the consulting offices as well as the radiodiagnostic department. This is worrisome since it strains the system and the departments working under this great pressure, generating long waiting lists, delays in performing tests and, consequently, delays in diagnoses and the initiation of treatments.

All of this has repercussions on the quality of the departments and on the diagnostic yield of the tests since it modifies their conditions of use and interpretation, alters the selection criteria and conditions some indications. This may, undoubtedly, have repercussions on the diagnostic value of the tests [8,9].

The radiodiagnostic departments are experiencing this overcrowding and often receive an excess number of requests for abdominal echographies which are not always justified and repeated tests are frequently requested with few clinical criteria. All of this means that physicians should carefully evaluate whether all the requests for echographies are truly justified and whether the result (for example, confirmation of cholelithiasis) would have repercussions on the therapeutic approach [10].

These circumstances have led to the creation of guidelines or recommendations to remit patients to the departments of diagnostic imaging in some countries such as the United Kingdom [11,12] and Spain. Thus, in Spain, the Spanish Society of Diagnosis by Imaging of the Abdomen (SEDIA) and the Spanish Society of Medical Radiology (SERAM) developed a document entitled "Criteria of Patient Remission to the Departments of Radiology (abdomen section)" adapted from the criteria of the European Commission and aimed at unifying criteria for remitting patients to radiology [13]. According to the experts a complementary test is useful when the result, whether positive or negative, aids in modifying the diagnostic therapeutic approach of the physician.

The main reasons for requiring remission regulations and criteria are:

1) To avoid the repetition of tests already performed.

2) To not request complementary tests which will most certainly not modify patient care.

3) To not request tests too early.

4) To not request tests with inadequate indications.

5) To provide the clinical information necessary and consider the questions which the diagnostic imaging tests should resolve.

6) Avoid excessive use of complementary tests.

These guidelines consider 280 clinical problems or situations depicted in four columns distributed as follows: the first column covers the clinical situation, the second indicates the most indicated diagnostic imaging tests possible, the third gives a recommendation as to whether the examination is adequate or not and the fourth column provides explanatory comments. The recommendations used are: indicated, specialized examination, not initially indicated, not systematically indicated and not indicated.

Therefore, to avoid any error in interpretation, the requests for imaging tests must be duly and legibly completed with a clear explanation of the reason for the request for the examination and provide sufficient clinical information for the radiodiagnostic specialist to understand the diagnostic approach or the problems which they aim to resolve with the radiological examination. Nonetheless, many specialists have perceived that this requisite is not always fulfilled and these aspects should be improved [10,13].

Some studies have analysed the requests for abdominal echographies remitted by primary care physicians to radiology departments and the main criteria analysed is the percentage of pathological alterations detected. In general, 30% of the echographies performed detected pathological alterations and state that the diagnostic performance could be improved if relevant clinical information were included in the requests [14]. In addition, the introduction and the use of guidelines or recommendations for the request for diagnostic imaging tests in general and particularly for echographies, have reduced the number of these requests by 25% to 30% [15]. Thus, in 2003, the Catalan Institute of Health, which belongs to the Government of Catalonia, ordered the development of guidelines of recommendations and criteria of indication for computerised tomography and magnetic resonance and their consequent application has had a positive repercussion in the sense of improvement in the indications of these tests and a reduction in the number of remissions [16].

In primary care the main reasons for requesting an abdominal echography are an alteration in the liver function tests, pain in the upper right quadrant, unspecific abdominal disturbances, control of chronic liver diseases, and control of biliary polyps and cysts. Of all these situations, the most appropriate for requesting an abdominal echography are for the screening of hepataocarcinoma in patients with chronic liver diseases [2], the presence of gallstones [5], the study of probable steatosis in patients with alterations in liver function tests [4,17] and the control of biliary polyps. It is not very useful in either the study of dyspepsias [18] or unspecific abdominal disturbances [19].

Apart from evaluating whether the request for the echography have sufficient information correct or not, it is important to analyse whether it is adequate or not [20,21]. We believe that this point is very important and needs the effort of both clinicians and radiologists to achieve a consensus of criteria when requesting echographies. It has been reported that the use of guidelines or recommendations reduces the percentage of requests and, in addition, the establishment of criteria regarding whether the indication is adequate or not, this demand would probably be further reduced, thereby improving the diagnostic yield. There are no studies in the literature evaluating the adequacy or not of echographies. However, some reports have evaluated the changes in therapeutic attitude of physicians based on the results of echographies and this is interpreted as good adequacy [22].

Along this line, one of the most important studies carried out in the Netherlands was a prospective cohort study undertaken from April 2003 to December 2004 in a total of 76 primary care physicians and 396 patients from three areas, The objectives were to quantify the rate of influence of positive and negative abdominal echography findings on the change in therapeutic attitude and management of the patients and evaluate the consequences of abdominal echography on the satisfaction of the patients. The most important results were a change in therapeutic attitude in 64% of the cases, a significant reduction in the number of consultations with specialists (from 45% to 30%) and an increase in the resolution of the patients from primary care (from 15% to 43%). To the contrary, this change was not evaluated by the patients [23].

## Objectives

### Main objective

To evaluate the adequacy of abdominal echographies requested by primary care physicians in our reference area (Maresme, province of Barcelona).

### Secondary objectives

1. Determine the quality of the requests for abdominal echographies.

2. Develop guidelines of recommendations and criteria of indication for abdominal echography with consensus among primary care physicans and specialists.

3. Evaluate the impact of the implementaction of these guidelines on the adequacy of the use of this procedure.

## Methods/Design

The study will be divided into two phases, one descriptive, retrospective evaluating the adequacy and quality of the requests for abdominal echographies, and the second evaluating the impact on the adequacy and quality of the requests for abdominal echographies through an intervention based on the recommendations and the criteria of indication of the abdominal echographies for primary care professionals. **The study has been approved by the Ethical Committee of Clinical Investigation, Jordi Gol i Gurina Foundation**.

### Study subjects

We will include all the abdominal echographies requested from January 2007 to April 2010 up to to sample size necessary by the primary care physicians of the health care centres of Arenys, Llavaneres, Premià, Vilassar de Mar, Vilassar de Dalt and 5 health care centres of the city of Mataró (Riera, Rocafonda, Cerdanya, Gatassa and Ronda Prim) remitted to the Department of Radiology of the CAP II of the Maresme, the diagnostic imaging centre of reference for these primary care teams.

The same number of requests for abdominal echographies from the same physicians will be evaluated after the implementation of guidelines of recommendations and criteria of indication of abdominal echography.

### Sample size

It has been calculated that 200 abdominal echographies are requested per month, therefore the 1067 echographies necessary should be collected within the foreseen study period (January 2007-April 2010), accepting an alpha risk of 0.05 for a precision of ± 3% in a bilateral contrast for an estimated proportion of adequacy of abdominal echographies of 50%. This sample size will also allow detection of an improvement of 7% or more in adequacy.

### Phase I (baseline study)

#### Variables

All the requests for abdominal echography will be analysed and the electronic clinical histories of the patients will be used to determine the following variables:

1. Personal data of the patient:

- Personal Identification Code

- Gender

- Date of birth

- Primary care centre which the patient attends

2. Visit-related data:

- Date of the consultation

- Physician

- Reason for the visit:

Abdominal pain-discomfort (epigastrium-upper right quadrant), biliary dyspepsia, unspecific abdominal disturbances, analytical examination, control of chronic liver disease, control of biliary polyps, control of biliar lithiasis, control of haemangiomas, control of hepatic cyst, control of renal lithiasis, control of renal cyst, others.

- Reason for the request for echography:

Alteration in liver function tests, abdominal pain (epigastric-right hipochondrium) biliary dyspepsia, repeated vomiting, unspecific abdominal disturbances, control of chronic liver disease, control of biliary polyps, control of biliary lithiasis, control of haemangiomas, control of hepatic cyst, control of renal lithiasis, control of renal cyst, others.

- Diagnostic approach

- Type of request: Ordinary or urgent

3. Clinical data of the patient:

a. History related to the test and/or possible disease:

- Obesity

- AHT

- Diabetes

- Dyslipemia

- Alcohol consumption

- Consumption of hypolipemiants

b. Anthropometric data:

- Weight (Kg)

- Height (cm)

- Body mass index (weight in Kg/height in m^2^)

c. Analytical determinations (in the last 3 months):

- Alanine aminotransferase (ALAT)

- Aspartate aminotransferase (ASAT)

- Gammaglutamyltranferase (GGT)

- Hepatitis B virus surface antigen (HBsAg)

- Hepatitis C virus antibodies

4. Data of the abdominal echography:

- Date

- Radiologist

- Results:

Normal, steatosis, biliary lithiasis, biliary polyps, calcifications or granulomas, alteration in hepatic structure, haemangioma, hepatic cyst, renal lithiasis, renal cyst, others.

5. Evaluation of the request for abdominal echography:

Quality:

- Very good: detailed clinical information of the reason why the patient attended the consultation and the physician also reports the diagnostic approach.

- Good: Sufficient clinical information on the reason for the consultation with no details of the same or the diagnostic approach.

- Not good: Scarce clinical information on the reason for the consultation.

- Regular: Generic information (for example: Lump, abdominal pain)

- Bad: No information.

### Phase IIa

Design of consensus guidelines on the use of abdominal echography clearly defining the criteria of adequate use of this diagnostic procedure. The consensus will be made by different experts, general practitioners, radiologists and gastroenterologists. The methodology to be used will be the consensus technique denominated "the nominal group technique" [24].

Registry of the adequacy of the abdominal echographies in phase I.

### Phase IIb

Diffusion of the recommendations and the criteria of indication of abdominal echography through meetings and sessions in the different centres with all the professionals therein and the edition of leaflets with the guidelines to give out to each physician of the different centres.

### Phase IIc

Evaluate the impact of the use of the guidelines on the adequacy of abdominal echographies, greater rationalisation of echography as a diagnostic test and the quality of the requests. To do this new 1067 abdominal echograhies from the same physicians will also be analysed and the same variables as those in phase I will be studied.

### Plan of analysis

The data will be introduced in a ACCESS database for statistical analysis.

Descriptive univariate analysis will be performed of the quantitative variables (percentiles, means and standard deviation) and the qualitative variables (frequency and percentages). Descriptive bivariate statistical analysis will also be carried out of the main variables (adequacy and quality of the echographies) and some of the secondary variables. To do this, the chi-square and Fisher exact tests will be used for comparison among the main qualitative variables and the secondary qualitative varibles and the Student's-t test and the non parametric Mann-Whitney tests will be used for the secondary quantitative variables. Statistical significance will be set at p < 0.05.

To compare the quality of the requests for echography and the adequacy of the abdominal echographies before and after the implementation of the guideline of recommendations and criteria of use of abdominal echograhies the chi-square test will be used since non paired data will be involved. Statistical significance will be determined at p < 0.05. Thus, the confidence interval will be 95%. The Stata version 11 statistical programme will be used to analyse the data.

### Study limitations

One of the limitations of this study may be the lack of a definition of adequacy of abdominal echographies. This will be defined once the initial field work has been finalised by analysing all the data and obtaining a consensus related to the criteria of adequacy of abdominal echographies among the different experts with the aim of providing plans of action.

Another limitation is that the information of some of the associated variables will be collected based on the registries of computerised clinical histories with the risk this implies of finding inexistent data. We will attempt to palliate this by proactively complementing the non reported data with the physician in charge of the cases.

The introduction of a control group has been considered but we believe that the presence of such a group could present a serie of limitations. The presence of this type of group would require the selection of either a specific number of physicians or different centres which would induce these to modify the remission criteria because of their having been selected. Some of the physicians or centres selected could also refuse to continue once the project has been initiated. In the short term no changes which could modify the medical criteria of action of the different professionals are expected in our health care system.

We are therefore before an important problem since abdominal echography is a very advantageous examination because of its innocuousness, low cost and diagnostic reliability. These advantages have led to increasing requests for abdominal echographies in primary care with the consequent rise in pressure on the radiodiagnostic departments. The importance of this project lies in determining whether the professionals in our reference area remit abdominal echographies well. On the other hand, it is also important to determine whether these requests are adequate or not. With the aim of establishing the latter, a work group will be created among general practitioners, gastroenterologists and radiologists. The results of this consensus and according to the results obtained we aim to create a guideline of recommendations to define the reasons and adequacy of abdominal echography and thereby avoid the undertaking of unnecessary tests, reduce costs and avoid overcrowding of the departments of radiodiagnosis.

## Competing interests

The authors declare that they have no competing interests.

## Authors' contributions

MaAALL, LCR, LRG, PTM, JDCC, JCS, JALL, ANP and GPB participated in the design of the study; LCR, MaAALL, LRG, SCC, JBS, MAP, ANP contributed to the coordination study; DMB, JDCC, CSG and JAC participated in the coordination of the echographies; GPB participated in the statistical calculations. All the authors have read, revised and approved the final manuscript.

## Pre-publication history

The pre-publication history for this paper can be accessed here:

http://www.biomedcentral.com/1471-230X/10/101/prepub

## References

[B1] Mittelstaedt CA Doyma Ecografía abdominal 1989 Barcelona 1 80

[B2] Forner A Vilana R Ayuso C Bianchi L Solé M Ayuso JR Boix L Sala M Varela M Llovet JM Bru C Bruix J Diagnosis of hepatic nodules 20 mm or smaller in cirrhosis: Prospective validation of the noninvasive diagnostic criteria for hepatocellular carcinoma Hepatology 2008 47 97 104 10.1002/hep.21966 18069697

[B3] Marn ChS Bree RI Silver TM Ultrasonography of liver. Technique and focal and diffuse disease Radiol Clin North Am 1991 29 1151 1170 1947039

[B4] Caballería Ll Auladell MA Torán P Miranda D Aznar J Pera G Gil D Muñoz L Planas J Canut S Bernad J Aubà J Pizarro G Aizpurua MM Altaba A Tibau A Prevalence and factors associated with the presence of non alcoholic fatty liver disease in an apparently healthy adult population in primary care units BMC Gastroenterol 2007 7 41 10.1186/1471-230X-7-41 17983472 PMC2151951

[B5] Puylaert JBCM Ultrasonography of acute abdomen: gastrointestinal conditions Radiol Clin N Am 2003 41 1227 1242 10.1016/S0033-8389(03)00120-9 14661668

[B6] Livraghi T Meloni F Di Stasi M Rolle E Solbiati L Tinelli C Rossi S Sustained complete response and complication rates after radiofrequency ablation of very early hepatocellular carcinoma in cirrhosis: Is resection still the treatment of choice? Hepatology 2008 47 82 89 10.1002/hep.21933 18008357

[B7] Wong SN Lin CJ Chen WT Cua IH Lin SM Combined percutaneous radiogrequency ablation and ethanol injection for hepatocellular carcinoma in high-risk locations Am J Roentgenol 2008 190 187 195 10.2214/AJR.07.2537 18287411

[B8] Mathers N Hodgkin P The Gatekeeper and the Wizard: a fairy tale BMJ 1989 298 172 173 10.1136/bmj.298.6667.172 2493843 PMC1835499

[B9] Liel Y Fraenkel N Use and misuse of thyroid ultrasound in the initial workup of patients with suspected thyroid problems referred by primary care physicians to an endocrine clinic J Gen Intern Med 2005 20 766 768 10.1111/j.1525-1497.2005.0124.x 16050890 PMC1490189

[B10] Díaz Rodriguez N La ecografía en atención primaria SEMERGEN 2002 28 376 384

[B11] Royal College of Radiologists Working Party Influence of Royal College of Radiologists guidelines on referral from general practice Br Med J 1993 306 110 111 10.1136/bmj.306.6870.110 PMC1676679 8435606

[B12] Board of Faculty of Clinical Radiology, The Royal College of Radiologists Guidance for the training in ultrasound of medical non-radiologists 1997 London: The Royal College of Radiologists

[B13] Criterios de Remisión de Pacientes a los Servicios de Radiología Area de Abdomen Realizados por la Sociedad Española para el diagnóstico por la imagen de abdomen (SEDIA). Sociedad Española de Radiología Médica (SERAM). Adaptación de los elaborados por la comisión europea. Madrid;Septiembre 2002

[B14] Connor SE Banerjee AK General practitioner requests for upper abdominal ultrasound: their effect on clinical outcome Br J Radiol 1998 71 1021 1025 10211061 10.1259/bjr.71.850.10211061

[B15] van Breuseghem I Geusens E Assessment of the appropriateness of requested radiological examinations for outpatients and the potential financial consequences of guideline application JBR-BTR 2006 89 8 11 16607870

[B16] Recomanacions i criteris d'indicació de tomografia computada I ressonància magnètica Institut Català de la Salut. Generalitat de Catalunya 2003 Departament de Sanitat I Seguretat Social 1 53

[B17] Browne RF Hamilton S Abdominal ultrasound in the evaluation of the asymptomatic patient with abnormal liver function tests Ir Med J 2004 97 49 50 15134270

[B18] Heikkinen M Räsänen H Färkkilä M Clinical value of ultrasound in the evaluation of dyspepsia in primary health care Scand J Gastroenterol 2005 40 980 984 10.1080/00365520510015845 16165712

[B19] van den Heuvel-Janssen HAM Borghouts JAJ Muris JWM Koes BW Bouter LM Knottnerus JA Chronic non-specific abdominal complaints in general practice: a prospective study on management, patient health status and course of complaints BMC Family Practice 2006 7 12 10.1186/1471-2296-7-12 16512926 PMC1420306

[B20] Lázaro P Fitch K Jovell A, Aymerich M Análisis del uso apropiado de la tecnología médica 1999 Monografía sobre Ciencias de la Salud: Academia de Ciencias Médicas de Cataluña y Baleares; Evidencia Científica y toma de Decisiones en Sanidad. Barcelona 185 200

[B21] Peiró S Meneu R Roselló ML Portella E Carbonell-Sanchís R Fernández C Lázaro G Llorens MA Martinez-Mas E Moreno E Ruano M Rincón A Vila M Validez del protocolo de evaluación del uso inapropiado de la hospitalización Med Clin 1996 107 124 129 8754481

[B22] Knop FK Stauning JA The benefits of diagnostic imaging in general practice Ugeskr Laeger 2006 168 794 798 16499845

[B23] Speets AM Hoes AW van der Graaf Y Kalmijn S de Wit NJ van Swijndregt AD Gratama JW Rutten MJ Mali WP Upper abdominal ultrasound in general practice: indications, diagnostic yield and consequences for patient management Fam Pract 2006 23 507 511 10.1093/fampra/cml027 16790453

[B24] Peiró S Portella E El grupo nominal en el entorno sanitario 1994 1a Quaderns de salut pública i administració de serveis de salut 1 31

